# Advances in Traditional Chinese Medicine for the treatment of hyperuricemia and associated diseases: pathogenesis, mechanisms, and future directions

**DOI:** 10.3389/fnut.2025.1663096

**Published:** 2025-09-11

**Authors:** Zhuo Huang, Bushuang Li, Ming-hui Bi

**Affiliations:** Xiamen Hospital of Traditional Chinese Medicine, Xiamen, Fujian, China

**Keywords:** hyperuricemia, Traditional Chinese Medicine, gout, cardiovascular diseases, renal disorders, personalized medicine, network pharmacology, nanotechnology

## Abstract

Hyperuricemia (HUA), characterized by elevated serum uric acid levels (>420 μmol/L), is a metabolic disorder linked to gout, cardiovascular diseases, renal disorders, diabetes, polycystic ovary syndrome (PCOS), and non-alcoholic fatty liver disease (NAFLD). Traditional Chinese Medicine (TCM) offers a holistic approach to HUA management, employing dialectical treatments to address underlying pathogenesis, reduce uric acid, mitigate inflammation, and protect organ function. This review synthesizes recent advances in TCM for HUA and its comorbidities, drawing from pharmacological studies of single herbs, compound formulas, and TCM pathogenesis theories. We introduce innovative strategies, including network pharmacology, metabolomics, personalized TCM diagnostics, and nanotechnology, to enhance therapeutic precision and efficacy. By integrating TCM's traditional wisdom—emphasizing balance among vital energies like “qi” (vital energy) and bodily fluids, with modern scientific methodologies, this review highlights potential risks, toxicities, and challenges in TCM application, aiming to improve patient outcomes in HUA and related diseases.

## 1 Introduction

Hyperuricemia (HUA) is a prevalent metabolic disorder defined by serum uric acid levels exceeding 420 μmol/L in men and 360 μmol/L in women, resulting from dysregulated purine metabolism or impaired uric acid excretion (1, 2). It is a major risk factor for gout, cardiovascular diseases, renal disorders, diabetes, polycystic ovary syndrome (PCOS), and non-alcoholic fatty liver disease (NAFLD) (3). The global prevalence of HUA is rising due to dietary shifts, obesity, and aging populations.

In modern medicine, HUA is managed with uric acid-lowering drugs such as allopurinol and febuxostat, which inhibit xanthine oxidase (XOD), or uricosurics like benzbromarone, which enhance uric acid excretion (4, 5). However, these treatments are associated with side effects, including hepatotoxicity, renal impairment, and gastrointestinal disturbances, and often fail to address comorbidities or prevent gout recurrence (6, 7). Moreover, patient adherence is low due to adverse effects and the chronic nature of the condition (8).

Traditional Chinese Medicine (TCM) offers a holistic alternative, viewing HUA as a condition of “ben xu biao shi” (deficiency in the root and excess in the manifestation), often linked to deficiencies in the spleen and kidney (organs associated with digestion, fluid metabolism, and vitality in TCM), damp-heat accumulation (a pathogenic factor resembling inflammation and fluid retention), phlegm-turbidity (mucus-like stagnation correlating to metabolic buildup), and blood stasis (impaired circulation akin to vascular dysfunction) (9). TCM employs single herbs and compound formulas to reduce uric acid levels, suppress inflammation, and protect organ function, with potentially fewer adverse effects compared to Western drugs in some studies (10, 11). However, challenges such as herb-drug interactions, variability in herb quality, and rare reports of hepatotoxicity must be considered. This review synthesizes findings from various sources, expands on TCM's therapeutic mechanisms, and introduces innovative approaches such as network pharmacology, metabolomics, personalized diagnostics, and nanotechnology to advance HUA treatment.

Despite these advantages, TCM faces limitations, including inconsistent evidence from clinical trials, potential toxicities from improper use, and the need for stronger backing for safety claims.

## 2 Methodology

This narrative review synthesizes recent advances in Traditional Chinese Medicine (TCM) for the treatment of hyperuricemia (HUA) and its associated diseases, focusing on pathogenesis, mechanisms, therapeutic approaches, and future directions. To ensure transparency and rigor, the review process followed a structured approach adapted from guidelines for narrative reviews, including elements of systematic methodology where applicable (e.g., explicit search strategies and quality appraisal) to minimize bias and improve reliability (12). Literature was selected through systematic searches conducted in major databases, including PubMed, Web of Science, China National Knowledge Infrastructure (CNKI), Wanfang Data, and VIP Database. Search terms included “hyperuricemia,” “gout,” “traditional Chinese medicine,” “Chinese herbal medicine,” “uric acid metabolism,” “xanthine oxidase,” and related combinations in both English and Chinese. The search covered publications from January 2000 to June 2025 to ensure inclusion of contemporary studies while building on foundational research. Manual hand-searching of reference lists from key articles and relevant journals (e.g., Journal of Ethnopharmacology, Chinese Journal of Integrative Medicine) was also conducted to identify additional sources. Duplicates were removed using reference management software (EndNote X9), with discrepancies resolved through discussion.

Inclusion criteria were: (1) peer-reviewed articles, clinical trials, experimental studies, or reviews addressing TCM's role in HUA pathogenesis, mechanisms, treatments, or innovations; (2) studies demonstrating clear methodologies, such as *in vitro*/*in vivo* experiments or randomized controlled trials; and (3) relevance to human health outcomes or molecular mechanisms. Exclusion criteria included: (1) non-peer-reviewed sources (e.g., conference abstracts or gray literature); (2) case reports or small-scale studies with fewer than 10 participants; (3) articles focused solely on Western medicine without TCM integration; and (4) duplicates or irrelevant topics. Initial searches yielded approximately 1,200 articles, which were screened by title and abstract, followed by full-text review. Ultimately, 138 references were selected based on quality, relevance, and contribution to the field, with an emphasis on high-impact journals and evidence-based findings to minimize bias. No formal meta-analysis was performed, as this is a narrative synthesis aimed at providing a comprehensive overview rather than quantitative pooling, given the heterogeneity in study designs (e.g., *in vitro* vs. clinical) and TCM-specific outcomes.

## 3 Mechanisms and associated diseases of hyperuricemia (HUA)

### 3.1 Uric acid metabolism and production

Uric acid is the final product of purine metabolism in humans, primarily synthesized in the liver through the enzymatic activity of xanthine oxidase (XOD). Purine nucleotides, derived from dietary sources or endogenous cellular turnover, are metabolized into hypoxanthine and xanthine, which XOD converts into uric acid. Approximately 70% of uric acid is excreted via the kidneys, with the remaining 30% eliminated through the intestines (13). The kidneys reabsorb about 90% of filtered uric acid, mediated by specific transporters, making renal handling a critical determinant of serum uric acid levels.

### 3.2 Mechanisms of hyperuricemia

HUA arises from an imbalance between uric acid production and excretion, leading to elevated serum uric acid levels (>6.8 mg/dL) (13). The key mechanisms include:

#### 3.2.1 Overproduction of uric acid

A primary driver of HUA is the overproduction of uric acid, which occurs when XOD activity is upregulated, accelerating the conversion of purine precursors (hypoxanthine and xanthine) into uric acid. Several factors contribute to this process. Genetic mutations or polymorphisms in genes such as *phosphoribosyl pyrophosphate synthetase (PRPS)* enhance uric acid synthesis by altering purine metabolism pathways. High-purine diets, including consumption of red meat, organ meats, shellfish, and certain fish (e.g., sardines, anchovies), increase purine load, thereby elevating uric acid production. Alcohol consumption, particularly beer and spirits, exacerbates this by promoting ATP degradation to purines and enhancing hepatic XOD activity, leading to higher uric acid levels (14). Additionally, high fructose intake, common in sweetened beverages, upregulates purine metabolism via the fructokinase pathway, further contributing to uric acid overproduction (15).

#### 3.2.2 Underexcretion of uric acid

A significant contributing factor to HUA is the dysregulation of uric acid transporters in the proximal tubules of the kidneys. Urate Transporter 1 (URAT1), encoded by the *SLC22A12* gene, facilitates uric acid reabsorption, with polymorphisms like rs475688 linked to decreased excretion and increased HUA risk (16). Similarly, Glucose Transporter 9 (GLUT9), encoded by *SLC2A9*, is a critical regulator of uric acid reabsorption, and its genetic variants are strongly associated with HUA and gout susceptibility (17). Organic Anion Transporters (OAT1 and OAT3), encoded by *SLC22A6* and *SLC22A8*, respectively, mediate uric acid secretion, but dysfunctional variants impair this process, contributing to elevated uric acid levels (18, 19). Additionally, the ATP-Binding Cassette Subfamily G Member 2 (*ABCG2*) transporter, encoded by the *ABCG2* gene, supports uric acid secretion in both kidneys and intestines; the Q141K polymorphism (rs2231142) is a well-established risk factor for HUA and gout (20). Other factors, including renal dysfunction, diuretics (e.g., thiazides, loop diuretics), and lactic acidosis, further inhibit uric acid excretion by competing for transporter activity, exacerbating HUA (21–24). These molecular and physiological disruptions highlight the complex interplay of genetic and environmental factors in HUA pathogenesis, necessitating targeted therapeutic strategies to enhance renal uric acid clearance.

#### 3.2.3 Uricase deficiency

Unlike most mammals, humans lack functional uricase, an enzyme that degrades uric acid into the more soluble allantoin. This evolutionary loss results in higher baseline uric acid levels in humans (typically 3.5–7.2 mg/dL) compared to other mammals (e.g., <2 mg/dL in rodents) (25, 26). Uricase deficiency, combined with dietary and genetic factors, predisposes humans to HUA (13).

### 3.3 Diseases associated with hyperuricemia

Elevated uric acid levels contribute to multiple pathological conditions through crystal-dependent and crystal-independent mechanisms (9). These include:

#### 3.3.1 Gout

Gout results from the deposition of monosodium urate (MSU) crystals in joints and soft tissues, triggering acute inflammatory responses (27, 28). MSU crystals activate the NLRP3 inflammasome, leading to interleukin-1β (IL-1β) release and neutrophil infiltration, causing gouty arthritis. Risk factors include obesity, hyperlipidemia, and alcohol consumption, which exacerbate HUA and crystal formation (29, 30).

#### 3.3.2 Cardiovascular diseases

HUA is an independent risk factor for cardiovascular diseases through multiple molecular mechanisms. In hypertension, HUA induces oxidative stress and endothelial dysfunction by reducing nitric oxide bioavailability and activating the renin-angiotensin system, resulting in elevated blood pressure (31, 32). Similarly, HUA promotes coronary heart disease by driving vascular smooth muscle cell proliferation and atherosclerotic plaque formation, increasing the risk of myocardial infarction (31, 32). Furthermore, elevated uric acid levels contribute to atrial fibrillation by promoting atrial remodeling and oxidative stress, which precipitate arrhythmic events (33).

#### 3.3.3 Renal disorders

HUA contributes significantly to renal pathology through multiple mechanisms. The deposition of monosodium urate (MSU) crystals in renal tubules triggers interstitial nephritis, leading to tubular obstruction and inflammation, which impair kidney function (34). Chronic HUA further exacerbates renal damage by inducing tubulointerstitial fibrosis and glomerulosclerosis, accelerating the progression of chronic kidney disease (CKD) (35). This relationship is bidirectional, as HUA both causes and results from renal dysfunction, creating a vicious cycle that amplifies kidney injury (21).

#### 3.3.4 Type 2 diabetes

HUA exacerbates insulin resistance by inducing oxidative stress and impairing glucose uptake in peripheral tissues, such as skeletal muscle and adipose tissue (36, 37). These molecular disruptions contribute to metabolic dysfunction, with elevated uric acid levels correlating with higher fasting glucose and HbA1c concentrations, thereby increasing the risk of type 2 diabetes (38–40). By promoting oxidative stress and disrupting insulin signaling pathways, HUA plays a pivotal role in the pathogenesis of diabetes, highlighting the need for integrated therapeutic strategies to address both uric acid levels and glucose metabolism.

#### 3.3.5 Polycystic ovary syndrome (PCOS)

HUA is closely associated with insulin resistance and hyperandrogenism in polycystic ovary syndrome (PCOS), exacerbating its metabolic and hormonal disturbances. Elevated uric acid levels promote oxidative stress and inflammation, which impair insulin signaling and amplify hormonal imbalances, contributing to metabolic dysfunction in PCOS patients (41, 42).

#### 3.3.6 Non-alcoholic fatty liver disease (NAFLD)

HUA promotes hepatic fat accumulation, contributing to non-alcoholic fatty liver disease (NAFLD) through distinct molecular mechanisms. Uric acid induces mitochondrial oxidative stress, which impairs fatty acid β-oxidation, leading to lipid accumulation in hepatocytes (43, 44). Additionally, uric acid upregulates lipogenic enzymes, such as sterol regulatory element-binding protein 1 (SREBP-1), driving hepatic steatosis and accelerating NAFLD progression (44, 45).

### 3.4 TCM pathogenesis

In TCM, HUA, though not explicitly named, is classified under conditions such as “*li jie*” (calendar joint pain, akin to arthralgia or gout-like symptoms), “*bi zheng*” (arthralgia syndrome, referring to obstructive pain syndromes), or “*tong feng*” (gout), reflecting its systemic and articular manifestations (46, 47). TCM attributes HUA to internal deficiencies, primarily of the spleen (associated with digestion and fluid transformation) and kidney (linked to filtration and essence storage), and external pathogenic factors, including wind (rapid onset symptoms), cold (constricting pain), dampness (heaviness and swelling), and heat (inflammation and redness) (48, 49). Spleen deficiency impairs water metabolism, while kidney deficiency hinders fluid and uric acid excretion, leading to the accumulation of damp-heat (a combination of fluid retention and inflammation) or phlegm-turbidity (mucus-like stagnation resembling metabolic deposits), which correlates with impaired uric acid clearance observed in modern studies (50). Excessive damp-heat stagnation obstructs “qi” and blood flow, manifesting as joint pain, swelling, and systemic inflammation, akin to gouty arthritis (49). Chronic HUA further promotes phlegm-turbidity and blood stasis, contributing to organ damage and comorbidities such as cardiovascular and renal diseases, mirroring molecular findings of uric acid-induced inflammation and fibrosis (48).

External pathogens, such as wind, cold, or dampness, exacerbate joint symptoms and systemic imbalances by invading the body and disrupting homeostasis (9). TCM categorizes HUA-related diseases into syndromes—such as damp-heat, phlegm-turbidity, or liver-kidney “yin” deficiency—guiding dialectical treatments tailored to individual patterns, which align with personalized medicine approaches (46, 51). This framework highlights TCM's holistic perspective, offering insights that complement molecular understanding of HUA pathogenesis. Recent integrative research using multi-omics techniques has further elucidated these TCM patterns, revealing that spleen deficiency correlates with disrupted purine metabolism and gut dysbiosis, impairing uric acid degradation, while kidney deficiency aligns with transporter dysregulation (e.g., URAT1 inhibition) and chronic kidney disease progression (52). External factors like damp-heat are supported by studies showing TCM extracts promoting UA excretion and repairing oxidative damage through multi-target effects, such as modulating immune-inflammatory responses (53). This convergence validates TCM's role in addressing HUA's root causes, paving the way for personalized therapies. While TCM theories align with modern findings, contradictions exist in clinical outcomes, where some studies show variable efficacy due to individual differences and a lack of standardization (54).

## 4 TCM monotherapy for hyperuricemia

TCM monotherapies target uric acid production, excretion, and associated symptoms in HUA, offering a safer profile with fewer side effects compared to Western drugs like allopurinol in preliminary studies (55); however, risks such as hepatotoxicity from high doses or contaminated herbs have been reported (56). Table 1 summarizes key TCM herbs, their active compounds, molecular mechanisms, uric acid reduction efficacy, and additional therapeutic effects, highlighting their multi-target approach for HUA management. Besides, these herbs demonstrate dose-dependent efficacy, targeting multiple pathways (XOD inhibition, transporter modulation, anti-inflammation) and offering a safer profile than allopurinol. Emerging 2024–2025 research highlights how these herbs exert multi-target effects, such as inhibiting UA reabsorption and promoting excretion via transporters like URAT1, while alleviating oxidative stress—aligning TCM's “clearing heat and dampness” with anti-inflammatory mechanisms in hyperuricemia models (57, 58).

**Table 1:** Key TCM single herbs for hyperuricemia: active compounds, mechanisms, and therapeutic effects.

**Herb (Latin name)**	**Active compounds**	**Mechanisms**	**Uric acid reduction**	**Additional effects**	**References**
Plantaginis Semen (*Plantago asiatica L.)*	Verbascoside, luteolin, flavonoids	Inhibits XOD; anti-inflammatory effects	20.37–50.10% (50–200 mg/kg)	Alleviates joint pain and swelling	(120, 121)
Polygonum cuspidatum *(Reynoutria japonica* Houtt.*)*	Flavonoids (e.g., resveratrol), stilbenes	Inhibits XOD; modulates URAT1, GLUT9, OAT3	25.58–43.41%	Reduces oxidative stress	(58, 122–124)
Yam Rhizome (*Dioscorea opposita* Thunb.)	Dioscin	Inhibits URAT1, promotes uric acid excretion	17.67–25.58% (30–300 mg/kg)	Relieves gouty arthritis symptoms	(125)
Coptis chinense *(Coptis chinensis* Franch.*)*, Phellodendron chinense *(Phellodendron chinense* Schneid.*)*	Berberine	Inhibits XOD; protects renal function	34.02–43.89% (individual), 56.43% (combined)	Enhances renal protection	(104, 105, 126)
Salvia miltiorrhiza *(Salvia miltiorrhiza* Bunge*)*	Salvianolic acid A	Inhibits XOD (IC50 = 654.49 μM)	12.11–22.03%	Mitigates vascular inflammation	(127, 128)
Turmeric (*Curcuma longa* L.)	Curcumin, derivatives	Inhibits XOD; protects renal and hepatic function	36.47–43.33%	Protects against renal/hepatic damage	(106, 107)
Honeysuckle (*Lonicera japonica* Thunb.), Chrysanthemum (*Chrysanthemum morifolium* Ramat.), Galangal (*Alpinia officinarum* Hance)	Flavonoids (e.g., quercetin, luteolin)	Inhibits XOD (up to 50% for Chrysanthemum at high concentrations)	N/A	Reduces inflammation	(129, 130)
Ginkgo Leaf (*Ginkgo biloba* L.), Gynostemma (*Gynostemma pentaphyllum* (Thunb.) Makino)	Flavonoids, gypenosides	Modulates ABCG2, OAT1/3; inhibits XOD	N/A	Improves insulin sensitivity, renal function	(131)
Chinese Violet (*Viola mandshurica* W. Becker), Corn Silk (*Zea mays* L.)	Flavonoids, polysaccharides	Promotes uric acid excretion without diuretic effects	N/A	Improves kidney function in gouty nephropathy	(132)

^*^Evidence in this table is predominantly from animal studies (rodent models).

Despite promising results, contradictions in efficacy exist; for instance, some animal studies show inconsistent uric acid reduction due to dosage variability (10). Potential toxicities include renal strain from diuretic herbs like Plantaginis Semen (*Plantago asiatica* L.) in long-term use (59), and allergic reactions to flavonoids in *Polygonum cuspidatum* (*Reynoutria japonica* Houtt.) (60). Challenges include herb standardization, as bioactive compound levels vary by source (61), and limited human trials to confirm safety over Western drugs (62). Future studies should address these to substantiate safety claims.

## 5 TCM compound formulas for hyperuricemia and related diseases

TCM compound formulas combine multiple herbs to address complex syndromes in HUA, leveraging synergistic effects to target uric acid metabolism, inflammation, and organ protection with fewer side effects than Western drugs. Recent advances demonstrate these formulas' efficacy in rodent HUA models through regulating purine metabolism and immune responses, with integrative studies confirming alignment between TCM dampness resolution and biomedical UA transporter modulation for enhanced safety and precision (58). Table 2 summarizes TCM compound formulas, their constituent herbs, molecular mechanisms, uric acid reduction efficacy, and additional therapeutic effects, highlighting their multi-target approach for managing HUA and its comorbidities. These formulas embody TCM principles like “jun chen zuo shi” (emperor-minister-assistant-courier herb roles) while aligning with biomedical synergy in pathway modulation.

**Table 2:** TCM compound formulas for hyperuricemia: constituent herbs, mechanisms, and additional therapeutic effects.

**Formula**	**Constituent herbs**	**Mechanisms**	**Additional effects**	**References**
Bixie Chubi Decoction	Yam rhizome (*Dioscorea opposita* Thunb.), Atractylodes (*Atractylodes macrocephala* Koidz.), Plantago (*Plantago asiatica* L.)	Down-regulates URAT1, up-regulates OAT1; suppresses TLR4/MyD88/NF-κB pathway	Alleviates gouty arthritis inflammation	(133)
Guizhi shaoyao zhimu Decoction	Cassia twig (*Cinnamomum cassia* (L.) J.Presl), Peony (*Paeonia lactiflora* Pall.), Anemarrhena (*Anemarrhena asphodeloides* Bunge)	Reduces uric acid and creatinine; improves joint symptoms	Significantly reduces pain scores in gout patients (human study)	(134)
Ermiao Pill	Atractylodes (*Atractylodes macrocephala* Koidz.), Phellodendron chinense (*Phellodendron chinense* Schneid.)	Regulates purine metabolism; reduces joint inflammation; recent multi-omics studies reveal links to gut microbiota shifts for damp-heat syndromes.	Targets damp-heat syndromes	(52, 135)
Compound Smilax Glabra	Smilax glabra (*Smilax glabra* Roxb.), Turmeric (*Curcuma longa* L.), Corn silk (*Zea mays* L.)	Enhances uric acid excretion	Reduces liver/kidney damage in HUA rat models	(136)
Liuwei Tongfeng Decoction	Cortex fraxini (*Fraxinus chinensis* Roxb.), Polygonum cuspidatum (*Reynoutria japonica* Houtt.), Rhubarb (*Rheum officinale* Baill.)	Inhibits XOD	Not specified	(137)
Qushi Dizhuo Decoction	Atractylodes (*Atractylodes macrocephala* Koidz.), Smilax glabra (*Smilax glabra* Roxb.), Salvia miltiorrhiza (*Salvia miltiorrhiza* Bunge)	Inhibits uric acid production; promotes excretion	Improves cardiovascular health	(137)

^*^Evidence in this table is predominantly from animal studies (rodent models).

While synergistic effects are highlighted, contradictions arise in meta-analyses showing high heterogeneity in trial outcomes, possibly due to poor blinding or small sample sizes (63). Toxicities may include liver enzyme elevation from berberine-rich formulas like Ermiao Pill (*Atractylodes macrocephala* Koidz. and *Phellodendron chinense* Schneid.) in susceptible patients, and challenges in quality control lead to contamination risks (64). Claims of superior safety require more robust RCTs comparing TCM to allopurinol directly (65).

## 6 TCM treatment for HUA-related diseases

### 6.1 Cardiovascular diseases

HUA is a significant risk factor for cardiovascular diseases, including coronary heart disease, hypertension, and atrial fibrillation, driven by oxidative stress, endothelial dysfunction, and vascular smooth muscle proliferation (66). In TCM, these conditions are attributed to “qi” deficiency (vital energy depletion), blood stasis (circulation impairment), and phlegm-turbidity (metabolic stagnation), which align with molecular disruptions such as inflammation and vascular remodeling (67). TCM offers targeted interventions to address these pathologies holistically (68). Buyang Huanwu Decoction, comprising *Astragalus* (*Astragalus membranaceus* (Fisch.) Bunge), *Angelica sinensis* (*Angelica sinensis* (Oliv.) Diels), and *Ligusticum* (*Ligusticum chuanxiong* Hort.), promotes “qi” and blood circulation (enhancing energy flow and vascular health), reducing uric acid levels, and mitigating cardiovascular risk factors (69). Danhong Injection, combining *Salvia miltiorrhiza* and Safflower, resolves blood stasis, enhances endothelial function, and reduces uric acid by 20–30%, offering protection against vascular complications (70). Similarly, Xuefu Zhuyu Decoction targets blood stasis and “qi” stagnation, effectively lowering hypertension and uric acid levels in HUA patients with cardiovascular comorbidities (71).

### 6.2 Gout

Gout, driven by MSU crystal deposition, manifests as acute joint inflammation or chronic tophi, resulting from hyperuricemia-induced crystal formation that triggers NLRP3 inflammasome activation and interleukin-1β release (13). In TCM, gout is classified into distinct syndromes based on its acute and chronic phases, guiding targeted treatments. In the acute phase, characterized by damp-heat or wind-damp-heat syndromes (inflammation with fluid retention or rapid-onset swelling), Qingre Lishi Decoction (containing *Plantago asiatica* L. and *Atractylodes macrocephala* Koidz.) and Canxiang Decoction reduce uric acid levels and inflammation, effectively alleviating pain and swelling by addressing damp-heat accumulation (72). In the chronic phase, marked by phlegm-turbidity or liver-kidney “yin” deficiency, Wuling Powder (containing Poria and Atractylodes) and Qufeng Huoluo Xiezhuo Decoction promote uric acid excretion, while Gui Shao Dihuang Decoction nourishes “yin” (restoring fluid balance) and reduces tophi formation, mitigating chronic joint damage (73). These TCM interventions target both uric acid metabolism and inflammatory pathways, offering a holistic approach to managing gout's acute and chronic manifestations, complementing molecular insights into MSU-driven pathology (54).

### 6.3 Renal disorders

In TCM, renal disorders are attributed to spleen-kidney deficiency (impaired digestion and filtration) and damp-turbidity accumulation (fluid and metabolic stagnation), which align with impaired uric acid clearance and inflammation observed in modern studies (74). TCM offers targeted interventions to mitigate these pathologies. Smilax glabra (*Smilax glabra* Roxb.) and Corn silk (*Zea mays* L.) promote uric acid excretion and protect renal tubules, reducing proteinuria and creatinine levels to preserve kidney function (75, 76).

Clerodendranthus spicatus (*Orthosiphon stamineus* Benth.) enhances uric acid clearance and improves glomerular filtration rate, offering therapeutic benefits in gouty nephropathy (77). Zhenwu Decoction (containing *Poria cocos* (Schw.) Wolf, *Atractylodes macrocephala* Koidz., *Paeonia lactiflora* Pall., *Rehmannia glutinosa* (Gaertn.) DC., and *Zingiber officinale* Roscoe) strengthens spleen and kidney function, reduces dampness (fluid retention), and protects against renal fibrosis, addressing both the root causes and manifestations of HUA-related renal damage (78).

### 6.4 Diabetes

In TCM, this comorbidity is termed “xiaoke tongfeng” (thirsting-wasting with gout-like pain), attributed to damp-heat and phlegm stasis, which align with molecular disruptions in glucose and uric acid metabolism (79). TCM offers targeted interventions to address these overlapping pathologies. Gynostemma [*Gynostemma pentaphyllum* (Thunb.) Makino] and *Sophora flower* (*Sophora japonica* L.) improve insulin sensitivity and reduce uric acid levels by 15–25%, mitigating metabolic dysfunction (80). Salvia miltiorrhiza (*Salvia miltiorrhiza* Bunge) and Astragalus [*Astragalus membranaceus* (Fisch.) Bunge] enhance glucose metabolism and protect pancreatic β-cells, addressing both HUA and diabetes through anti-inflammatory and antioxidant effects (81). Liuwei Dihuang Pill [containing *Rehmannia glutinosa* (Gaertn.) DC., *Dioscorea opposita* Thunb., *Cornus officinalis* Siebold & Zucc., *Paeonia suffruticosa* Andrews, *Poria cocos* (Schw.) Wolf, and *Alisma plantago-aquatica* L.] nourishes the kidney “yin,” improving insulin sensitivity and reducing uric acid, offering a holistic approach to managing this dual pathology (82).

### 6.5 Polycystic ovary syndrome (PCOS)

PCOS is attributed to kidney deficiency (vitality depletion) and phlegm-damp stagnation (metabolic and fluid buildup), which align with molecular disruptions in insulin signaling and hyperandrogenism (83). TCM offers targeted interventions to address these overlapping pathologies. Cangfu Daotan Decoction, Atractylodes (*Atractylodes macrocephala* Koidz.) and Poria [*Poria cocos* (Schw.) Wolf], resolves phlegm-dampness, reduces uric acid levels, and improves ovulatory function by mitigating metabolic dysfunction (84). Heqi Powder [containing *Bupleurum chinense* DC., *Angelica sinensis* (Oliv.) Diels, and others] regulates liver “qi” (smoothing emotional and energy flow) and clears dampness, addressing HUA and enhancing fertility in PCOS patients by balancing hormonal and metabolic pathways (85).

### 6.6 Non-alcoholic fatty liver disease (NAFLD)

In TCM, NAFLD is attributed to phlegm-stasis (metabolic obstruction), reflecting metabolic and inflammatory disruptions (86). TCM employs herbs like Salvia miltiorrhiza (*Salvia miltiorrhiza* Bunge), Poria [*Poria cocos* (Schw.) Wolf], and Hawthorn (*Crataegus pinnatifida* Bunge) to resolve phlegm-stasis, reduce hepatic lipid accumulation, and mitigate inflammation (87). Formulas such as Yinchenhao Decoction (containing *Artemisia scoparia* Waldst. & Kitam., *Gardenia jasminoides* J.Ellis, and *Rheum officinale* Baill.) lower uric acid levels and liver enzymes, improving NAFLD outcomes by targeting both metabolic and inflammatory pathways (88). These TCM interventions offer a holistic approach, complementing molecular insights into HUA-driven NAFLD pathogenesis by addressing uric acid accumulation and hepatic lipid metabolism (89).

## 7 Innovative approaches in TCM for HUA

### 7.1 Network pharmacology and metabolomics

Network pharmacology has transformed TCM research by elucidating herb-compound-target interactions, offering a systems-level understanding of therapeutic mechanisms for HUA (90). For instance, studies of Polygonum cuspidatum (*Reynoutria japonica* Houtt.) and Bixie Chubi Decoction (*Dioscorea opposita* Thunb., *Atractylodes macrocephala* Koidz., *Plantago asiatica* L.) demonstrate multi-target effects on xanthine oxidase (XOD), urate transporter 1 (URAT1), and inflammatory pathways such as TLR4/MyD88/NF-κB, revealing how TCM formulas modulate uric acid metabolism and inflammation—bridging traditional “multi-herb synergy” with biomedical network analysis (91). For a concrete example, in the TCM formula Ermiao Pill, the active compound berberine (derived from Phellodendron chinense) demonstrates strong molecular docking affinity to xanthine oxidase (XOD), with binding energies around −10 kJ/mol, effectively inhibiting uric acid production as validated *in vitro* and in hyperuricemia models (92). Another instance involves Simiao Powder, where compounds like quercetin target key inflammatory proteins such as IL-1β and TNF, modulating the NLRP3 inflammasome pathway to reduce gout-related inflammation (93). Metabolomics complements this approach by identifying key biomarkers, such as purine, tryptophan, and tyrosine metabolites, which guide the optimization of formulas like Ermiao Pill for enhanced efficacy and specificity (94). Specific biomarkers include L-tyrosine and L-phenylalanine, which are downregulated in HUA patients, indicating disruptions in amino acid metabolism that affect thyroid hormone synthesis and contribute to metabolic disorders like insulin resistance. Additionally, arachidonic acid serves as a biomarker, showing reduced levels linked to heightened inflammation and cardiovascular risks in HUA (95). Looking forward, these approaches hold immense potential to advance TCM for HUA management. They can predict herb-drug interactions to improve safety profiles, mitigating risks of adverse effects when combining TCM with conventional therapies (90). Additionally, network pharmacology can uncover synergistic effects in compound formulas, enabling the design of more effective herb combinations tailored to specific HUA syndromes. Metabolomics-driven insights will further optimize dosages and herb selections by correlating biomarker profiles with TCM syndromes, facilitating personalized treatment strategies. Recent literature on TCM-modern integration highlights network pharmacology's role in drug innovation, predicting synergies for HUA by mapping herb targets to UA pathways, including novel atavistic approaches like mRNA-mediated uricase expression in hepatocytes (52, 96). Future research should integrate these tools with artificial intelligence and clinical trials to validate multi-target mechanisms, refine TCM formulations, and establish standardized protocols for global adoption (97). This convergence of TCM wisdom and cutting-edge omics technologies promises to enhance therapeutic precision, improve patient outcomes, and bridge traditional and modern medicine in addressing HUA and its comorbidities.

Despite promising results, challenges include database inaccuracies and incomplete data on TCM compounds, leading to biased predictions. Limited technological advancements hinder novel target identification, and the complexity of TCM formulas complicates validation in clinical settings (98). Metabolomics faces issues with biomarker specificity in HUA, as metabolic profiles vary by patient factors like diet, potentially causing inconsistent outcomes (99). Future efforts should focus on standardized databases and multi-omics integration to overcome these barriers.

### 7.2 Personalized TCM diagnostics—enhancing TCM's dialectical approach with genetic and metabolic profiling for hyperuricemia

Traditional Chinese Medicine (TCM) employs a dialectical approach (“bian zheng lun zhi”), customizing treatments to an individual's unique syndrome patterns, such as damp-heat (inflammation with retention) or spleen-kidney deficiency (digestive and filtrative weakness), rather than targeting the disease alone. This holistic, patient-centered method draws on a practitioner's expertise and clinical observation, offering flexibility but sometimes lacking objective precision.

Integrating genetic and metabolic profiling into TCM provides a scientific basis to enhance its personalized approach. For hyperuricemia (HUA), these advanced techniques identify specific genetic variants and metabolic alterations driving the condition. By complementing TCM's syndrome-based framework, this data enables precise, evidence-based interventions, strengthening the efficacy of tailored treatments.

A prime example involves polymorphisms in the URAT1 gene (*SLC22A12*), a uric acid transporter in the kidneys. Patients with certain URAT1 variants, such as rs475688, exhibit impaired uric acid excretion, exacerbating HUA (16). TCM herbs like Yam rhizome (*Dioscorea opposita* Thunb.) and Plantago (*Plantago asiatica* L.) are traditionally used for their diuretic and uric acid-lowering effects, aligning with “promoting water metabolism” in TCM (100). Genetic profiling can confirm that these herbs are particularly beneficial for patients with URAT1 polymorphisms by targeting the underlying molecular mechanism, thus refining herb selection and improving outcomes.

Machine learning models, trained on datasets combining TCM syndrome patterns with omics data (genomics, metabolomics), hold transformative potential for HUA treatment:

° **Predicting Optimal Herb Combinations:** By analyzing a patient's genetic and metabolic profile, machine learning can recommend herb combinations tailored to their specific needs, minimizing trial-and-error and enhancing efficacy.
° **Correlating TCM Syndromes with Genetic Markers:** These models can identify links between TCM syndromes (e.g., kidney deficiency) and genetic variants (e.g., *SLC2A9* or *SLC22A12*), improving diagnostic precision and aligning traditional classifications with molecular evidence.
° **Developing Personalized Treatment Plans:** Machine learning can integrate HUA severity, comorbidities (e.g., diabetes, non-alcoholic fatty liver disease), and patient-specific data to design treatment plans that optimize dosages, herb selections, and therapeutic strategies.

This fusion of TCM's dialectical approach with cutting-edge science bridges ancient wisdom and modern precision medicine. It offers a data-driven path to more effective, individualized HUA treatments, reducing side effects and improving patient outcomes. The approach also sets a precedent for applying similar integrations to other chronic conditions addressed by TCM. Future advancements in genomics and artificial intelligence could further refine this model, fostering collaborations between TCM practitioners and researchers. Large-scale studies validating these correlations and the development of accessible AI tools for clinical use will be key to realizing its full potential.

Implementation faces ethical issues in genetic testing, such as informed consent and potential discrimination based on HUA risk profiles (101). Data privacy concerns arise from handling sensitive omics data, with risks of breaches in TCM databases lacking robust regulations (102). Limited access to genetic profiling in resource-poor settings exacerbates inequities, and variability in TCM practitioner expertise hinders standardization (103). Addressing these requires ethical guidelines and secure data frameworks.

### 7.3 Nanotechnology for TCM delivery

TCM utilizes bioactive compounds like curcumin (from *Curcuma longa* L., or turmeric, used in TCM for “invigorating blood”) and berberine (from *Coptis chinensis* Franch., known for “clearing heat”) to manage chronic conditions such as HUA (104–107). However, their therapeutic efficacy is often hindered by poor bioavailability due to low water solubility and limited gastrointestinal absorption. Compounds like curcumin are rapidly degraded, while berberine undergoes extensive metabolism, posing challenges for consistent delivery in HUA treatment, where sustained uric acid reduction and inflammation control are critical (108).

Nanotechnology offers innovative solutions through nanoparticle-based delivery systems, such as liposomes, micelles, and polymeric nanoparticles. These systems encapsulate hydrophobic TCM compounds, protecting them from degradation, enhancing solubility, and improving absorption across biological barriers. By increasing blood concentrations and prolonging therapeutic effects, nanotechnology ensures more effective management of HUA, addressing both uric acid metabolism and inflammation.

For HUA, nanotechnology enables precise targeting of affected tissues, such as the kidneys, where uric acid is excreted, and inflamed joints, where gout-related damage occurs (109). Surface-modified nanoparticles can concentrate in renal tissue, while the enhanced permeability and retention (EPR) effect allows accumulation in joints, reducing local inflammation (110). This targeted approach enhances efficacy and minimizes systemic exposure, improving treatment outcomes.

Nanoparticle types offer distinct advantages for TCM delivery. Liposomes, biocompatible lipid vesicles, carry both water- and fat-soluble compounds, with liposomal curcumin showing improved stability (111). Micelles excel at solubilizing hydrophobic drugs like berberine (112), while polymeric nanoparticles, such as PLGA, provide controlled release, making them versatile for tailored formulations (113). These systems allow lower doses to achieve therapeutic effects, reducing side effects like gastrointestinal irritation and improving patient compliance (114).

A notable example is nano-encapsulated curcumin, which has demonstrated a 10-fold increase in bioavailability in animal models, alongside enhanced anti-inflammatory and antioxidant effects (115). These improvements suggest that smaller doses could effectively manage HUA-related inflammation, offering a model for other TCM compounds. However, challenges remain in optimizing formulations for human use and confirming safety and efficacy through clinical trials.

Key challenges include scalability and translation from bench to clinic, with high production costs and regulatory hurdles for TCM-nano hybrids (116). Potential toxicities from nanomaterials (e.g., accumulation in organs) require long-term safety studies, especially for chronic HUA treatment. Variability in TCM extract quality complicates nano-formulation standardization. Future directions involve clinical trials to validate efficacy and safety.

Looking ahead, nanotechnology holds transformative potential to elevate TCM into a precise and potent therapeutic option for HUA and other chronic conditions. Continued research and clinical validation will be key to overcoming current limitations, bridging traditional TCM wisdom with modern delivery innovations to enhance patient outcomes.

### 7.4 AI-driven TCM optimization for HUA

Artificial intelligence (AI) is revolutionizing TCM by analyzing vast datasets of syndromes, herb compositions, and clinical outcomes to optimize treatment for HUA. By merging centuries-old TCM wisdom, such as syndrome patterns like “damp-heat,” with modern computational techniques, AI enhances therapeutic precision and efficacy in managing HUA.

AI algorithms, including natural language processing and machine learning, excel at identifying novel herb combinations for HUA. By sifting through extensive TCM literature, historical clinical data, and patient records, these algorithms uncover synergistic herbal interactions that traditional formulas may overlook. For instance, network pharmacology predicts how herbs interact with molecular targets and pathways involved in uric acid metabolism, enabling the design of innovative combinations with improved efficacy and fewer side effects (90, 91, 94, 97, 100).

Personalized medicine is advanced through AI's ability to predict treatment responses based on individual patient profiles. Supervised learning models, trained on datasets encompassing genetic profiles, lifestyle factors, and medical histories, identify patterns and biomarkers linked to successful HUA outcomes. This predictive power allows practitioners to customize herbal prescriptions and treatment plans, enhancing therapeutic effectiveness and patient satisfaction.

The integration of omics data—genomics, proteomics, and metabolomics—with TCM diagnostics represents a groundbreaking leap. AI processes these complex datasets to map traditional TCM syndromes, such as dampness-heat or “qi” deficiency, to specific molecular profiles associated with HUA. This fusion validates TCM's holistic approach with scientific evidence, improves diagnostic precision, and enables biologically informed, personalized treatments, bridging traditional practices with modern biomedical science.

Ethical issues in AI-based personalized medicine include bias in algorithms trained on limited datasets, potentially overlooking diverse populations in HUA treatment (117). Data privacy concerns are prominent, as AI relies on patient genetic and metabolic data, risking breaches without stringent protections like GDPR equivalents for TCM (102). Trust challenges arise from AI's “black box” nature, complicating TCM diagnosis validation, and data scarcity in TCM hinders model accuracy (118). Solutions involve transparent AI models and ethical frameworks.

By transforming TCM into a more precise, evidence-based practice, these AI-driven applications offer new strategies for effectively managing HUA. The synergy of ancient knowledge and cutting-edge technology paves the way for redefined therapeutic approaches, promising improved outcomes for patients with HUA and its comorbidities.

## 8 Limitations and challenges in TCM application

While Traditional Chinese Medicine (TCM) shows promise in managing hyperuricemia (HUA), its limitations—such as variability in herb quality, inconsistent clinical evidence, and potential herb-drug interactions—must be addressed for safe and effective integration. Herb quality varies due to factors like geographical origin, harvest timing, processing, and contamination risks (e.g., heavy metals or pesticides), resulting in inconsistent bioactive levels, for example, batch-to-batch fluctuations in resveratrol content in Polygonum cuspidatum can diminish xanthine oxidase (XOD) inhibition and clinical reliability, highlighting the need for enhanced standardization through quality control databases and authentication protocols (12). Clinical trials often suffer from methodological flaws, including small samples, lack of randomization, and poor reporting, as seen in reviews of TCM for gout, where efficacy appeared superior but evidence was weak due to bias; TCM's individualized approach further clashes with standardized RCTs, necessitating pragmatic designs and CONSORT extensions. Herb-drug interactions, such as berberine from *Coptis chinensis* inhibiting cytochrome P450 enzymes or *Polygonum cuspidatum* amplifying anticoagulants, raise safety concerns with urate-lowering drugs like allopurinol, demanding pharmacovigilance and monitoring (119). Ultimately, overcoming these hurdles through rigorous research, quality assurance, and interaction studies will bolster TCM's holistic benefits in HUA management.

## 9 Conclusions and future directions

TCM offers a holistic and effective approach to managing HUA and its comorbidities, with single herbs and compound formulas targeting uric acid metabolism, inflammation, and organ protection. Herbs like Plantaginis Semen (*Plantago asiatica* L.), *Polygonum cuspidatum* (*Reynoutria japonica* Houtt.), and compound formulas like Bixie Chubi Decoction (*Dioscorea opposita* Thunb., *Atractylodes macrocephala* Koidz., *Plantago asiatica* L.) demonstrate significant efficacy, often surpassing Western drugs in safety profiles. Innovations such as network pharmacology, metabolomics, personalized diagnostics, nanotechnology, and AI-driven optimization promise to enhance TCM's therapeutic precision and efficacy, bridging concepts like “qi” and “blood stasis” with biomedical pathways.

Future research should focus on:

- Safety and Toxicity Studies: Investigate potential risks, including herb-induced toxicities and contradictions in efficacy through post-marketing surveillance.
- Balanced Integration: Address challenges like standardization and evidence quality to support safety claims.
- Clinical Trials: Conducting large-scale, randomized controlled trials to validate TCM efficacy and safety across diverse populations.
- Omics Integration: Combining genomics, proteomics, and metabolomics to identify HUA-specific biomarkers and optimize TCM formulations.
- Personalized Medicine: Developing AI-driven tools to tailor TCM treatments based on individual genetic, metabolic, and syndrome profiles.
- Nanotechnology: Exploring nano-delivery systems to improve TCM compound bioavailability and target specificity.
- Global Collaboration: Integrating TCM with Western medicine through international research consortia to standardize protocols and expand clinical applications.

By merging TCM's holistic principles with cutting-edge technologies, we can advance the prevention and treatment of HUA and its associated diseases, ultimately improving patient outcomes and quality of life.
